# Selinexor *versus* doxorubicin in dedifferentiated liposarcoma PDXs: evidence of greater activity and apoptotic response dependent on p53 nuclear accumulation and survivin down‐regulation

**DOI:** 10.1186/s13046-021-01886-x

**Published:** 2021-03-01

**Authors:** Valentina Zuco, Sandro Pasquali, Monica Tortoreto, Silvia Brich, Stefano Percio, Gian Paolo Dagrada, Chiara Colombo, Roberta Sanfilippo, Calogero Lauricella, Mrinal Gounder, Rihan El Bezawy, Marta Barisella, Angelo Paolo Dei Tos, Paolo Giovanni Casali, Alessandro Gronchi, Silvia Stacchiotti, Nadia Zaffaroni

**Affiliations:** 1 Molecular Pharmacology Unit, Department of Applied Research and Technological Development, Fondazione IRCCS Istituto Nazionale Tumori, Via Amadeo 42, 20133 Milan, Italy; 2 Sarcoma Service, Department of Surgery, Fondazione IRCCS Istituto Nazionale Tumori, via Venezian 1, 20133 Milan, Italy; 3 Department of Pathology, Fondazione IRCCS Istituto Nazionale Tumori, via Venezian 1, 20133 Milan, Italy; 4 Adult Mesenchymal Tumor and Rare Cancer Unit, Department of Cancer Medicine, Fondazione IRCCS Istituto Nazionale Tumori, via Venezian 1, 20133 Milan, Italy; 5 Molecular Pathology Unit, Ospedale Niguarda Ca’ Grande, Milan, Italy; 6 Sarcoma Medical Oncology and Early Drug Development, Memorial Sloan Kettering Cancer Center, 1275 York Avenue, 10065 New York, NY USA; 7 Department of Medicine, University of Padua School of Medicine, Via Giustiniani 2, 35128 Padua, Italy; 8 Department of Biomedical and Clinical Sciences L. Sacco, University of Milan, Via Grassi 74, 20157 Milan, Italy

**Keywords:** Dedifferentiated liposarcoma, PDX, Primary cell culture, XPO1, Selinexor, Doxorubicin, Survivin

## Abstract

**Background:**

Dedifferentiated liposarcoma (DDLPS), a tumor that lacks effective treatment strategies and is associated with poor outcomes, expresses amplified MDM2 in the presence of wild-type p53. MDM2 ubiquitination of p53 facilitates its XPO1-mediated nuclear export, thus limiting p53 tumor suppressor functions. Consequently, nuclear export is a rational target in DDLPS. We directly compared the antitumor activity of the first-in class XPO1 inhibitor selinexor and doxorubicin, the standard front-line therapy in sarcomas, in DDLPS patient-derived xenografts (PDXs) and primary cell lines.

**Methods:**

Drug activity was assessed in three PDXs (and two corresponding cell lines) established from the dedifferentiated component of primary untreated retroperitoneal DDLPS with myogenic (*N* = 2) and rhabdomyoblastic (*N* = 1) differentiation from patients who underwent surgery. These models were marked by amplification of *MDM2*, *CDK4* and *HMGA2* genes.

**Results:**

Selinexor was moderately active in the three PDXs but achieved greater tumor response compared to doxorubicin (maximum tumor volume inhibition: 46–80 % vs. 37–60 %). The PDX harboring rhabdomyoblastic dedifferentiation showed the highest sensitivity to both agents. PDX response to selinexor and doxorubicin was not associated with the extent of *MDM2* and *CDK4* gene amplification. Interestingly, the most chemosensitive PDX model showed the lowest extent of *HMGA2* amplification. Selinexor was also more efficient than doxorubicinin in inducing an apoptotic response in PDXs and cell lines. Consistently, an increased nuclear accumulation of p53 was seen in all selinexor-treated models. In addition, a time-dependent decrease of survivin expression, with an almost complete abrogation of the cytoplasmic anti-apoptotic pool of this protein, was observed as a consequence of the decreased acetylation/activation of STAT3 and the increased ubiquitination of nuclear survivin.

**Conclusions:**

Selinexor showed a moderate antitumor activity in three DDLPS PDXs, which was, however, consistently higher than doxorubicin across all different models regardless the extent of MDM2 amplification and the histological differentiation. The depletion of survivin protein seems to significantly contribute to the induction of apoptosis through which selinexor exerts its antitumor activity.

## Background

Well differentiated (WD)/dedifferentiated (DD) liposarcoma (LPS) is the most frequent soft-tissue-sarcoma arising in the retroperitoneal space, accounting for approximately 50 % of all retroperitoneal sarcomas [1]. WDLPS is a low-grade disease composed of adipocytic cells that may recur locally, while DDLPS is a more aggressive non-lipogenic malignancy characterized by multifocal recurrences and, less frequently, distant site metastasis. Among others, DDLPS may undergo myogenic and rhabdomyoblastic heterologous differentiation, which conveys an aggressive phenotype to the tumor, mainly evident when rhabdomyoblastic traits are acquired [2]. Recent studies suggest that WDLPS and DDLPS divergently evolve from a common precursor harboring 12q13–q15 chromosomal sub-region amplification that results in the abnormal expression of genes such as *MDM2*, *CDK4* and *HMGA2*, which are the drivers of oncogenic transformation [3]. DDLPS has an overall more complex genomic profile than WDLPS, which may sustain its more aggressive phenotype, although specific drivers of the DDLPS distinct evolutionary pattern are to be clarified.

Surgery remains the primary treatment for localized DDLPS, but approximately 40 % of newly diagnosed patients will eventually die from advanced disease and, in particular, from local inoperable relapse. When surgery fails to achieve local tumor control, few effective therapeutic options are currently available [4]. No prospective trials focusing on doxorubicin-based chemotherapy have been conducted in DDLPS, while retrospective studies on anthracycline containing regimens have shown a limited antitumor effect [5, 6]. A higher degree of activity is seen when doxorubicin is combined to ifosfamide, which in the daily practice can also be used as monotherapy with a high-dose regimen [7]. Other options in further lines are represented by trabectedin or eribulin [8]. However, in all cases the antitumor effect of these anti-cancer agents remains limited.

The poor response of DDLPS to conventional systemic chemotherapy emphasizes the need for novel actionable targets and new therapies [9]. Indeed, the rarity of DDLPS, the heterogeneous clinical course of the disease, which is also determined by different patterns of differentiation, and the limited availability of translatable experimental models able to properly recapitulate clinical tumor biology and response to treatment strongly impair the development of innovative and effective treatments.

DDLPS harboring MDM2 amplification usually expresses wild-type p53. MDM2 ubiquitination of p53 facilitates its XPO1-mediated nuclear export, thus limiting p53 tumor suppressor functions [10]. Consequently, nuclear export is a rational target in DDLPS. Indeed, the first-in-class XPO1 inhibitor selinexor has shown activity in established cell line-based preclinical models of soft tissue sarcoma, including DDLPS [11–13], and a phase III randomized trial of selinexor versus placebo in patients with metastatic DDLPS (ClinicalTrials.gov identifier: NCT02606461) has recently completed the accrual [14]. A prospective study comparing selinexor and doxorubicin, the standard front-line therapy for metastatic DDLPS, has not been conducted.

In this study, we report the development and characterization of three patient-derived xenografts (PDXs), obtained from primary, treatment naïve, retroperitoneal DDLPS displaying myogenic and rhabdomyoblastic heterologous differentiation, which have been used to directly compare the antitumor activity of selinexor and doxorubicin, the standard front-line therapy in sarcomas. Results are reported herein.

## Materials and Methods

### Assessment of ***XPO1 ***gene expression in DDLPS clinical samples

Frozen tissues of the WD component, the DD component and the normal fat from each DDLPS were collected from 15 patients with a retroperitoneal, primary, untreated DDLPS. Specular formalin-fixed paraffin-embedded (FFPE) sections of each tumor were assessed for histology confirmation and quality check by a sarcoma-dedicated pathologist. Total RNA was extracted from frozen tissues with an RNeasy mini kit (Qiagen, Hilden, Germany). Gene expression profiles were assessed using the GeneChip Clariom S Human Arrays platform. Raw data was normalized according to the RMA algorithm of *oligo* Bioconductor package [15]. *XPO1* differential expression was estimated using the non-parametric Kruskal-Wallis statistic among the three tissue types. Nemeyi post-hoc test was used to assess significant differences (0.05 as threshold) in pair-wise comparisons. All analyses were performed using the computing microenvironment R.

The dataset generated in the study was deposited in the GEO database (GSE159659).

### Development of PDXs and patient‐derived cell lines

Each DD component of DDLPS clinical samples suitable for mouse implantation (based on dimension and tumor cellularity) was obtained from retroperitoneal primary tumors of three patients with retroperitoneal, treatment-naïve, MDM2 positive, FNCLCC (Fédération Nationale des Centres de Lutte Contre Le Cancer) grade 3 DDLPS. Myogenic and rhabdomyoblastic differentiation were defined as previously described [2]. Briefly, a DDLPS was characterized as having a myogenic differentiation when at least one myogenic immunohistochemistry marker among actin 14A, desmin and caldesmin was detected in > 10 % of neoplastic cells, while the presence of a positive immunostaining for myogenin was required to confirm the presence of rhabdomyoblastic differentiation.

A human tumour (LS-GD-1) showed histopathological and immunohistochemical features of rhabdomyoblastic heterologous dedifferentiation, as it contained rhabdoid cells and was positive for desmin, actin 1A4 and myogenin staining. The other two tumours displayed features of myogenic dedifferentiation, being LS-BP-1 positive for actin 14A and LS-BZ-1 positive for desmin. The use of patient material to generate PDXs and corresponding cell lines from consented patients was authorized by Fondazione IRCCS Istituto Nazionale Tumori (Milan, Italy) (INT) Ethics Committee (Project approval code: INT 139/17).

For establishment of PDX models, fresh tumor specimens were collected immediately after surgical resection, aseptically dissected and cut into small fragments (~ 3 mm^3^). At least 3 fragments were grafted subcutaneously into the right flank of 6-week old female CB17/lcr-Prkdc^scid^ (SCID) mice (Charles River, Calco, IT). The lag time for tumor growth after the first inoculum ranged from 45 to 68 days for the different models. Tumor growth was followed by biweekly measurement of tumor diameters with a Vernier calliper. Tumor volume (TV) was calculated according to the formula: TV (mm^3^) = d^2^ × D/2, where d and D are the shortest and the longest diameters, respectively. Tumors were transplanted into other mice when reached a TV of 600 mm^3^. A PDX was considered established after the third passage in mice and based on growth characteristics. The 3 models used in the study are part of a DDLPS PDX panel, consisting of 6 models we generated from the DD component of the 15 DDLPS (engraftment rate: 40 %) above mentioned.

Tumor fragments of established PDX models were engrafted into CD1-Foxn1^nu^ (nude) mice (Charles River) for preclinical pharmacology studies. SCID and nude mice were maintained in a pathogen-free facility where temperature and humidity were kept constant, and had free access to food and water.

To establish DDLPS cell lines, surgical tumor samples were cut into pieces of ~ 1 mm^3^ and enzymatically digested with collagenase (200 U/mL) (Sigma, St. Louis, MO, USA) for 3 h at 37°C. Cells were resuspended in DMEM supplemented with 10 % fetal bovine serum to inactivate collagenase (Merck) and applied to a cell strainer (100 µm, Corning), centrifuged at 500×g for 5 minutes and resuspended in DMEM supplemented with 10 % fetal bovine serum. Tumor cell lines were then propagated in DMEM F-12 medium (Lonza, Treviglio, Italy), supplemented with 10 % fetal bovine serum and maintained in a 37°C humidified 5 % CO2 incubator.

The origins of both PDXs and corresponding cell lines were authenticated through microsatellite analysis by the AmpFISTR Identifiler PCR amplification kit (Applied Biosystems, PN4322288, Foster City, CA, USA).

### Characterization of PDX models

Four micrometer sections of FFPE tumor tissue, obtained from sacrificed mice when tumor nodules were about 400 mm^3^, were stained with hematoxylin and eosin (H&E) for morphological evaluation and immunostained with MDM2 (anti-MDM2, Ab-1, mouse, MAb, IF2, #OP46, San Diego, CA, USA; dilution 1:40), CK AE1/AE3 (clone AE1-AE3, mouse, Dako, Santa CA, USA; dilution 1:100), EMA (clone E29, mouse, Dako; dilution 1:250), myogenin (clone F5D, mouse, Ready-to-use, Dako), actin (clone 1A4, mouse, Dako; dilution 1:250), desmin (clone D33, mouse, Dako; dilution 1:400), H3 trimethyl K27 (Ab H3K27me3, Clone C36B11, Cell Signaling, Beverly, MA, USA; dilution 1:400) and Ki67 (Ab Ki67, Clone Mib1, #GA626 Dako; dilution 1:400) antibodies for further characterization. Immunohistochemical (IHC) analysis was performed at room temperature on the Dako Autostainer Link 48 AS480 (Agilent, Santa Clara, CA, USA), as previously described [12]. Ki67 labeling index (number of Ki67-positive nuclei/overall number of nuclei x 100) was quantified using ImageJ 1.47q software (). FISH assay was carried out to assess *MDM2* gene amplification in the DDLPS clinical tumors and corresponding PDX models using ZytoLight SPEC MDM2/CEN 12 Dual Color Probe **(**ZytoVision GmbH. Bremerhaven Germany**)**.

*MDM2*, *CDK4*, *HMGA2* copy number variation was assessed by droplet digital PCR (ddPCR) on genomic DNA extracted from PDX tissue sections using Maxwell® CSC FFPE DNA AS1350. Twenty µl ddPCR reaction mixture was loaded into the Bio-Rad DG8 droplet generator cartridge that was then placed into the QX200 droplet generator. The generated droplets were manually transferred to an Eppendorf 96-well PCR plate that was then heat-sealed with a pierceable foil in the PX1 PCR Plate Sealer, and then amplified on the ProFlex PCR system. The thermal-cycling conditions were: 95°C for 10 min, 40 cycles of 94°C for 30 s, 60°C for 1 min, 98°C for 10 min and a final step at 4°C. After amplification, the 96-well PCR plate was loaded on QX200 droplet reader and ddPCR data were analyzed with QuantaSoft analysis software version 1.7.4.

### Evaluation of drug activity

Nude mice were randomized to receive either selinexor or doxorubicin which were administered when xenografts were approximately 150 mm^3^. Eight mice for each experimental group were used. The treatment dose and schedule for each drug were selected from the literature (Table 1). After dilution in sterile water, doxorubicin (Adriblastina, Pfizer Italia) was administered intravenously (*i.v.*) every 7 days for three times (q7d x 3). Selinexor (Selleck Chemicals, USA) was prepared as previously described [16] and delivered orally (*p.o.*) twice a week for 8 times (q3-4d x 8). Drug treatment activity was assessed in terms of TV inhibition percentage (TVI%) in treated *versus* control mice and expressed as TVI% = 100-[(mean TV treated/mean TV control) x 100]. Treatment toxicity was determined in terms of body weight loss and lethal toxicity.

**Table 1:** Pharmacological treatments and tumor responses of DDLPD PDX models

Treatment	Route	Schedule	Dose (mg/kg)	Max TVI % (day) ^a^			Tumor growth delay (days)^b^		
				**LS-GD-1**	**LS-BZ-1**	**LS-BP-1**	**LS-GD-1**	**LS-BZ-1**	**LS-BP-1**
**Doxorubicin**	*i.v.*	q7d×3	4	60 (49)^*^	40 (17)^*^	37 (46)^*^	20	5	16
**Selinexor**	*p.o.*	q3/4d×8	10	80 (49)^**^	55 (14)^*^	46 (55)^*^	> 46	7	23

^a^ Maximum tumor volume inhibition (TVI) % in treated versus control mice. In parentheses, the day on which it was assessed. ^*^P < 0.05, ^**^P < 0.005 versus control tumors

^b^ The tumor growth delay is calculated as the median time (days) in excess required for the tumor of the drug treated groups mice to reach 1000 mm^3^ compared to the diluents treated mice

 In vivo drug-based experiments were authorized by the Italian Ministry of Health according to the national law (Project approval code: 234/2018-PR) and in compliance with international policies and guidelines.

The growth inhibitory effect of selinexor and doxorubicin, singly administered at concentrations ranging from 1.0 nM to 1.0 µM for 72 h, was assessed by cell counting using a particle counter (Beckman Coulter, Luton, UK). The results were expressed as the number of adherent cells in treated samples compared with control samples. The in vitro drug activity was assessed in terms of concentrations able to inhibit cell growth by 50 % (IC_50_).

### Western blotting

Lysates were obtained from frozen PDX tumors collected immediately after (and, when indicated, after 12 days from) the end of mouse treatment with selinexor or doxorubicin as well as from DDLPS cell lines at different intervals after exposure to different concentrations of each drug. Nuclear and cytosolic fractions were obtained from DDLPS cells using NE-PER™ nuclear and cytoplasmic extraction reagents (#78,835; Thermo Fisher Scientific) following the manufacturer’s protocol.

For the assessment of the ubiquitinated form of survivin, the nuclear fraction of the protein was immunoprecipitated with the anti-human survivin antibody (#ab469; Abcam) and eluted as described [17]. Total/fractioned cellular lysates and immunoprecipitates were separated by SDS-PAGE, transferred onto nitrocellulose membranes and incubated with primary monoclonal antibodies: anti-MDM2 (#86,934, Cell Signaling Technology), anti-HMGA2 (#5269, Cell Signaling Technology), anti-cleaved CCP32 (#9661, Cell Signaling Technology), anti-STAT3 (#4904; Cell Signaling Technology), anti-acetyl-STAT3 (#2523; Cell Signaling Technology), anti-HDAC2 (#5113; Cell Signaling Technology), anti-LC3B (#2775 Cell Signaling), anti-survivin (#ab469; Abcam), anti-XPO1 (#ab24189; Abcam), anti-LC3B (#2775 Cell Signaling Technology), anti-ubiquitin (#ab7780, Abcam), anti-α-tubulin (T5168, Merck), anti-p53 (sc-126, Santa Cruz Biotechnology, CA, USA), anti-CDK4 (sc-601, Santa Cruz Biotechnology), anti-p16 (sc-6579, Santa Cruz Biotechnology) and anti-β-actin (A2066, Merck). Band intensities were quantified by scanning films and processing image intensities with the ImageJ 1.47v Software.

### Apoptosis analysis

Apoptosis was assessed in tumors excised from untreated and drug-treated mice using the SignalStain® Apoptosis IHC Detection Kit (Cell Signaling), which detects the presence of cleaved caspase-3, according to the manufacturer’s instructions. Sections were counterstained with hematoxylin. Number of caspase-3 positive cells/overall number of nuclei x 100 was quantified using ImageJ 1.47v Software.

Floating and adherent cells were harvested 72 h after treatment and processed for apoptosis evaluation with In Situ Cell Death Detection Kit, Fluorescein (#11,684,795,910, Merck), according to manufacturer’s instructions, and analyzed by BD™ Accuri C6 (Becton Dickinson, Mountain View, CA, USA). A specific software (CellQuestPro, Becton Dickinson) was used to estimate the percentage of apoptotic cells.

### Statistical analysis

Analyses by two-sided Student’s t-test were performed using the GraphPad Prism software, version 4.0 (GraphPad Prism Inc., San Diego, CA, USA). A *P* value of ≤ 0.05 was considered statistically significant.

## Results

### ***XPO1*** is over-expressed in clinical DDLPS samples

**Fig. 1:**
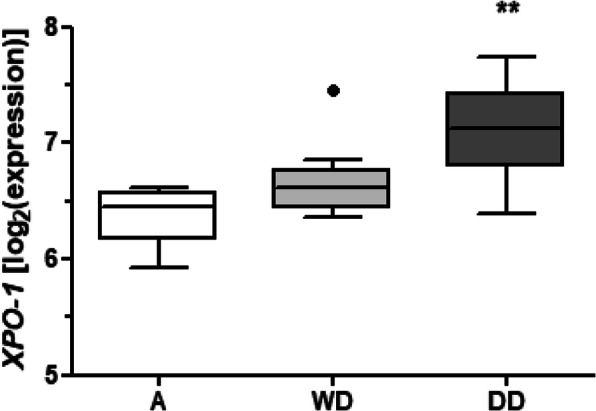
XPO1 expression in paired samples, including normal adipose tissue (A), WD component (WD) and DD component (DD), obtained from the retroperitoneal primary tumors of 15 treatment-naïve DDLPS patients. ^*^P < 0.05, ^**^P < 0.005

Gene expression profiles comparatively assessed on paired samples **–**including DD component, WD component and normal adipose tissue**–** obtained from 15 retroperitoneal primary tumors of treatment-naïve DDLPS patients showed a significantly increased expression of *XPO1* in DD and WD compared to healthy tissue (Fig. 1). These data confirmed a previous observation indicating a significantly higher *XPO-1* expression in DDLPS compared to lipoma samples [13].

This finding, together with *MDM2* amplification [3], strongly supports XPO1-mediated nuclear export as a rational target in DDLPS.

### PDX and cell model characterization

**Fig. 2:**
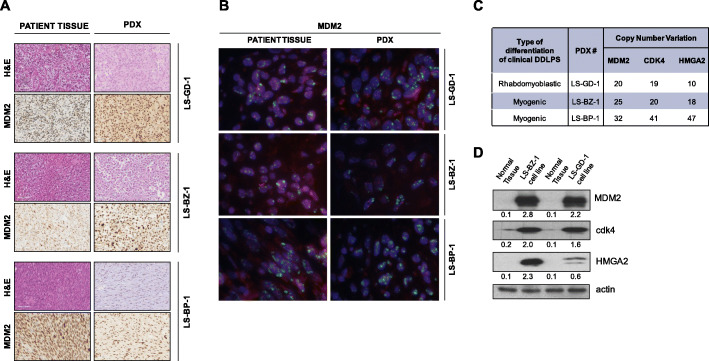
Characterization of patient-derived DDLPS models. **a**, Representative pictures of PDX models and corresponding clinical tumors. The histology was assessed on hematoxylin and eosin (H&E)-stained slides. MDM2 amplification was detected at the protein level by MDM2 immunostaing. **b**, FISH analysis: Spectrum Orange labeled Chromosome 12 centromere and Spectrum Green labeled MDM2. High level of *MDM2* amplification with clustering of gene copies are observed in both patient tissues and PDXs. **c**, Quantification of *MDM2, CDK4* and *HMGA2* copy number variation in PDXs by ddPCR. **d**, Assessment of MDM2, CDK4 and HMGA2 protein expression in cell lines by western blotting. A representative blot of three independent experiments is shown. For each protein, band intensity was quantified using Image J normalized to loading control reported below

Patient-derived tumor samples used for the generation of PDXs were consistent with the histopathological diagnosis of DDLPS (Fig. 2A). Two human tumors showed myogenic dedifferentiation (LS-BZ-1 and LS-BP-1) and a tumor showed rhabdomyoblastic dedifferentiation (LS-GD-1). The histomorphology of PDX models was consistent with the paired human samples (Fig. 2A). Immunophenotypic profile of models differed from the paired clinical tumours as LS-BP-1 and LS-BZ-1 PDXs lost expression of the myogenic markers Actin 14A and desmin, respectively. Detailed morphological and immunohistochemical characterization of PDXs is provided in Table 2.

**Table 2:** Histomorphology and immunophenotypic profile of DDLPS PDX models

	LS-BZ-1	LS-GD-1	LS-BP-1
***Human tumor***	*DDLPS, G3, myogenic dedifferentiation*	*DDLPS, G3, rhabdmyoblastic dedifferentiation*	*DDLPS, G3, myogenic dedifferentiation*
**H&E**	Spindle cells and epitheliod cells	Spindle cells organized in fascicles, and rhabdoid cells	Spindle cells with small nuclei
**CK1**	Negative	Negative	Negative
**CK3**	Negative	Negative	Negative
**EMA**	Negative	Negative	Negative
**Myogenin**	Negative	Positive, diffuse	Negative
**Actin 1A4**	Negative	Positive, focal	Negative
**Desmin**	Negative	Positive, diffuse	Negative
**H3K27me3**	Positive	Positive	Positive
**MDM2**	Positive	Positive	Positive

All clinical samples and corresponding PDXs were marked by *MDM2* gene amplification, as detected by IHC (Fig. 2A) and by FISH (Fig. 2B), and over-expressed MDM2 protein compared to normal cells (Fig. 2 C). Quantitative analysis of gene copy number by ddPCR showed a copy number gain for *MDM2*, *CDK4* and *HMGA2* genes in all PDX models, although to a variable extent (Fig. 2 C). Consistently, the two patient-derived cell lines were characterized by an enhanced expression of MDM2, CDK4 and HMGA2 proteins compared to the corresponding normal adipose tissues, as detected by western blotting (Fig. 2D).

### Selinexor is more active that doxorubicin and induces apoptotic response in all PDX and cell models

**Fig. 3:**
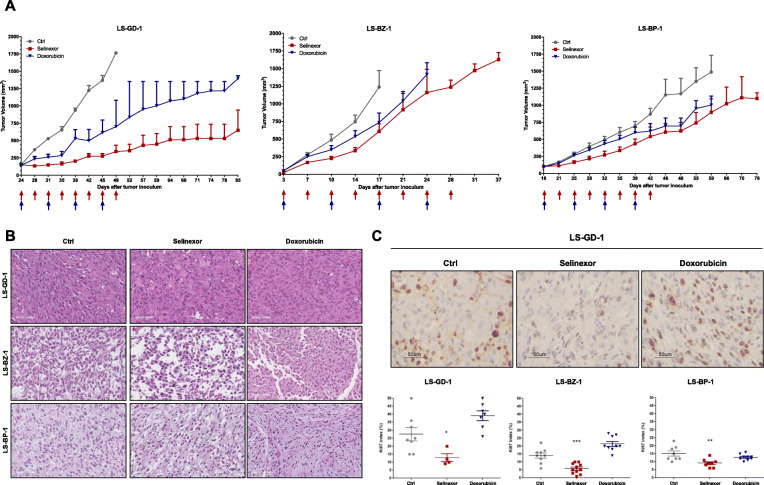
Antitumor activity of selinexor and doxorubicin in DDLPS PDXs. **a**, Growth curves report the average tumor volume (±S.E.M.) in control and drug-treated animal groups (8 mice/group). The arrows in the figure indicate when drugs were administered. **b**, Pathologic evaluation of tumors obtained from untreated (Ctrl) and selinexor- or doxorubicin-treated mice at the end of treatment. **c**, Ki67 immunostaining of tumors obtained from untreated (Ctrl) and selinexor- or doxorubicin-treated mice at the end of treatments (upper panel) and quantification of Ki67 index (lower panel). Two tumors for each experimental group were analyzed. The symbols reported in the lower panel represent counted fields. ^*^P < 0.05, ^**^P < 0.01, ^***^P < 0.005

In DDLPS animal models, selinexor and doxorubicin as single agents induced a tumor growth delay which was more pronounced for the XPO1 inhibitor than the anthracycline in all PDXs (maximum TVI%: 46–80 % vs. 37–60 %) (Fig. 3a; Table 1). The difference between selinexor and doxorubicin antitumor activity was particularly evident in the LS-GD-1 model, as also indicated by the significantly increased time for selinexor-treated animals to reach 1000 mm^3^ tumor burden compared to doxorubicin-treated and untreated animals (Table 1). This PDX model, which is characterized by rhabdomyoblastic dedifferentiation, was the most sensitive to both drugs.

The extent of selinexor and doxorubicin activity did not seem to be influenced by the level of *MDM2* and *CDK4* gene amplification. Interestingly, the most chemosensitive PXD model displayed the lowest extent of *HMGA2* amplification (Table 1; Fig. 2 c). No sign of toxicity, in terms of tumor weight loss and occurrence of toxic death, was registered with either drug.

*In vitro* cytotoxicity experiments carried out on DDLPS cell models showed that doxorubicin was active at lower concentrations than selinexor. The two cell models exhibited a comparable sensitivity to the anthracycline (IC_50_ values: 45 ± 16 nM and 46 ± 9 nM for LS-GD-1 and LS-BZ-1, respectively). Interestingly, consistent with *in vivo* data, LS-GD-1 cells displayed a higher susceptibility to selinexor than LS-BZ-1 cells (IC_50_ values: 116 ± 54 nM and 187 ± 59 nM, respectively).

Histopathological evaluation of drug-treated tumors excised from mice immediately after the end of treatment with selinexor or doxorubicin showed no marked differences at the morphological level, but only a slight and focal reduction in cellularity with an increased loose stroma in post-selinexor PDXs (Fig. 3b). The proliferation rate, as detected by Ki67 index, was significantly lower in selinexor-treated compared to untreated tumors in all PDXs. Conversely, doxorubicin exposure did not appreciably affect cell proliferation of any model (Fig. 3 c).

**Fig. 4:**
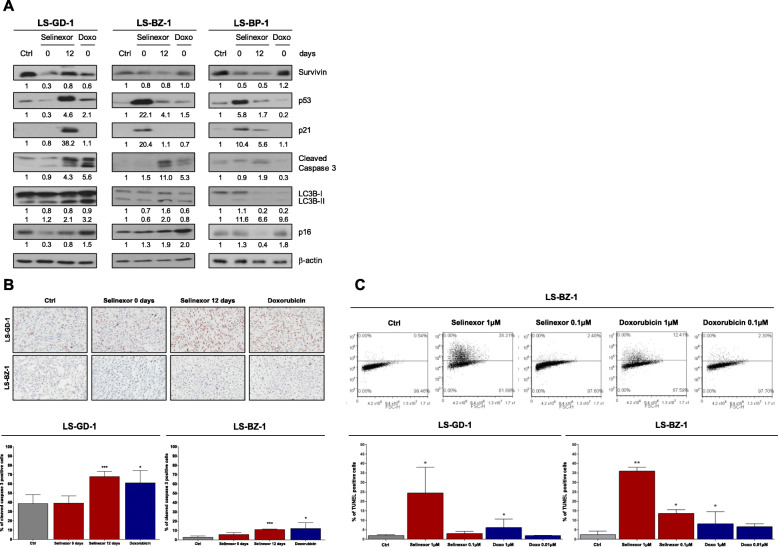
Induction of apoptosis by selinexor and doxorubicin in DDLPS models. **a**, Western blot analysis of survivin, p53, p21, and cleaved caspase 3 on tumors obtained from untreated (Ctrl) and selinexor- or doxorubicin-treated mice at different intervals from the end of treatment. The expression of autophagy (LC3B) and senescence (p16) markers was also assessed. A representative blot of three independent experiments is shown. For each protein, band intensity was quantified using Image J normalized to loading control reported below and referred to respective untreated control. The band intensity of LC3B-I and LC3B-II were quantified separately (above and below, respectively). **b**, Cleaved caspase-3 immunostaining of tumors obtained from untreated (Ctrl) and selinexor- or doxorubicin-treated mice at the end of treatments (upper panel) and quantification of the percentage of cleaved caspase-3 positive cells (lower panel). **c**, Flow cytometric assessment of TUNEL-positive cells after 72 h exposure to equimolar concentration of drugs in DDPLS cells (upper panel) and quantification of TUNEL-positive cells (lower panel). Results represent the mean values ± SD of 3 independent experiments. ^*^P < 0.05, ^**^P < 0.01, ^***^P < 0.005

Western blot analysis, which was performed at the end of treatment with each drug and also after 12 days for selinexor, revealed that the XPO1 inhibitor **–**but not doxorubicin**–** reduced the expression of the anti-apoptotic protein survivin and that, in 2 out of 3 PDX models, such a decrease was still appreciable 12 days after the end of treatment (Fig. 4a). Selinexor also induced an increased abundance of p53 protein, mainly appreciable at the end of treatment in two PDX models and after 12 days in the third model (Fig. 4a). In LS-BZ-1 and LS-BP-1 models, the over-expression of p53 was paralleled by an enhanced accumulation of p21/CDKN1A protein (Fig. 4a).

The induction of apoptosis, assessed in terms of cleaved caspase-3 expression by western blotting, was observed in all selinexor-treated PDXs, although to a very different extent, and was mainly appreciable 12 days after treatment. Caspase-3 cleavage was also detected at the end of doxorubicin treatment, though in LS-GD-1 and LS-BZ-1 models (Fig. 4a). Consistent results were obtained when the presence of cleaved caspase-3 was detected by immunohistochemistry (Fig. 4b).

In DDLPS cell models, flow cytometric detection of TUNEL-positive cells indicated a dose-dependent induction of apoptosis by both drugs, although the extent of the apoptotic response was consistently greater for selinexor than doxorubicin (Fig. 4b).

A slightly increased abundance of the autophagy marker LC3B-II was only observed 12 days or immediately after treatment with selinexor and doxorubicin, respectively, in the LS-GD-1 PDX, while a slightly enhanced expression of the senescence-associated marker p16 was appreciable after treatment with doxorubicin in all models (Fig. 4a).

#### Selinexor induces survivin down‐regulation by inhibiting STAT3 acetylation and promoting nuclear survivin ubiquitination

**Fig. 5:**
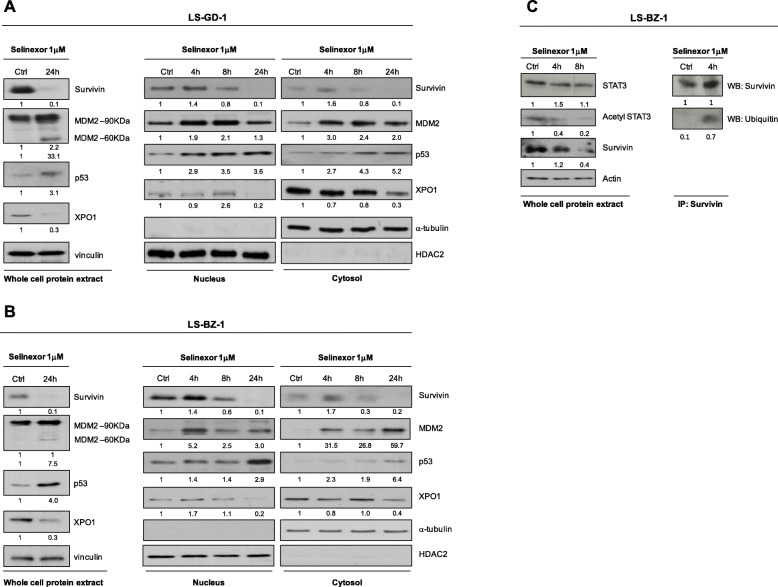
Effects of selinexor on the expression and subcellular localization of survivin. Western blot analysis of survivin, MDM2, p53 and XPO1 in whole protein extracts (left panel) and fractionated protein extracts (right panel) of LS-GD-1 cells **a**, and LS-BZ-1 cells **b**, at different intervals of exposure to selinexor. **c**, Western blot analysis of STAT3 and acetyl-STAT3 expression on whole protein extracts (left panel) and survivin ubiquitination in protein nuclear fraction (right panel) of LS-BZ-1 cells at different intervals of exposure to selinexor. Representative blots of three independent experiments are shown. For each protein, band intensity was quantified using Image J normalized to loading control reported below and referred to respective untreated control

The effect of drug treatment was assessed in more details in the two patient-derived DDLPS cell lines. Western blot analysis carried out on the whole protein extract showed that, consistently with *in vivo* findings, a 24 h exposure to 1 µM selinexor caused an enhanced accumulation of p53 protein in both cell models (Fig. 5a, b). Interestingly, drug treatment also induced the expression **–**mainly appreciable in the LS-GD-1 model (Fig. 5A,B)**–** of MDM2-p60, a MDM2 fragment generated by caspase-3-mediated cleavage, which loses the ring domain and, consequently, cannot target p53 to proteasome degradation [18]. Moreover, an almost complete abrogation of XPO1 and survivin expression was observed in both cell models following selinexor exposure (Fig. 5a, b).

The assessment of protein sub-cellular localization indicated that the increased expression of p53 in selinexor-treated cells was mainly ascribable to their enhanced nuclear accumulation (Fig. 5a, b). As regards survivin, whose sub-cellular localization is known to determine protein function [19], selinexor induced an early nuclear accumulation appreciable at 4 h, which was followed by a progressive decline of the protein in the nucleus and paralleled by a time-dependent reduction of cytoplasmic protein levels in both cell models (Fig. 5a, b).

Based on a previous report indicating that in triple-negative breast cancer cells the inhibition of XPO1 by selinexor was able to repress *BIRC5*/*survivin* gene transcription by inhibiting STAT3 acetylation and blocking STAT3 binding to the *survivin* promoter [20], we assessed STAT3 protein expression and acetylation in LS-BZ-1 cells. Western blot showed a time-dependent decrease of acetyl-STAT3 levels (Fig. 5 c), suggesting that survivin inhibition is related, at least in part, to post-translational modifications of its transcriptional activator also in DDLPS cells. Since we and others previously showed that the forced retention of survivin in the nucleus promotes its clearance by the ubiquitin-proteasome proteolytic pathway [21, 22], we checked whether selinexor might cause the ubiquitination of survivin nuclear fraction. Western blot results on nuclear survivin immunoprecipitates indicated that exposure of LS-BZ-1 cells to selinexor caused protein ubiquitination (Fig. 5 c). This finding suggests that proteasome-dependent degradation of survivin might concur to the overall reduction of protein expression induced by selinexor also in DDLPS cells.

## Discussion

In this preclinical study, we directly compared the activity of the XPO1 inhibitor selinexor and doxorubicin, the standard front-line medical therapy in sarcomas, on in-house developed DDLPS PDXs and corresponding cell lines. The results showed a moderate antitumor activity of selinexor, which was, however, consistently higher than doxorubicin in the different PDXs, irrespective of the extent of *MDM2* amplification and the heterologous differentiation subtype, although the most robust evidence of a superior activity of the XPO1 was observed in the PDX model showing rhabdomyoblastic differentiation. The depletion of anti-apoptotic survivin protein, consistently observed in *in vivo* and *in vitro* models, seemed to significantly contribute to the induction of apoptosis through which selinexor exerts its antitumor activity.

PDXs are robust preclinical models which retain the main characteristics of clinical tumors [23]. In this study, we found that, although our DDLPS PDXs maintained the histological features as well as the amplification of driver genes, which characterize the originating tumors, two of them do not express some of the immunophenotypic features of the corresponding clinical tumors. This finding could possibly reflect the sampling of DDLPS which are characterized by tumor heterogeneity, as indicated by differences in histomorphology, which may also reflect in genetic heterogeneity [24].

PDXs are more reliable than cell line-derived xenografts in predicting drug response [23]. Based on this premise, they are particularly useful for directly comparing standard-of-care agents and newer therapies in rare tumors, especially when prospective comparative clinical trials are lacking or challenges related to disease rarity question their feasibility. In this context, we already reported the consistency between preclinical data obtained on PDXs of ultra-rare sarcomas, such as solitary fibrous tumor and epithelioid sarcoma, and clinical results concerning the activity of different cytotoxic, anti-angiogenic and epigenetic agents [25–28]. The preclinical data showing effectiveness of the targeted drugs axitinib and pazopanib in our PDX models of solitary fibrous tumors were also instrumental to design new successfully conducted prospective phase II clinical trials [29, 30].

Previous studies on DDLPS established cell models showed the antiproliferative effect of selinexor and, in some instances, *in vitro* data were corroborated by *in vivo* evidence of antitumor activity of this drug in xenografts [11, 13]. This is the first report showing the effect of the XPO1 inhibitor on DDLPS patient-derived models, which also take into account the heterologous differentiation of PDXs as well as the extent of *MDM2*, *CDK4* and *HMGA2* gene amplification. Differently from a reported study indicating that doxorubicin-treated DDLPS cells demonstrated variable *in vitro* sensitivity based on baseline *MDM2* expression levels [31], we found that PDX response to selinexor and doxorubicin was not affect by the extent of *MDM2* and *CDK4* gene amplification. Interestingly, the most chemosensitive PDX model displayed the lowest extent of *HMGA2* amplification. A higher susceptibility to selinexor, but not doxorubicin, was also observed in the LS-GD-1 cell line that shows the lower HMGA2 protein expression.

Recent studies reported the involvement of *HMGA2* in the chemoresistant phenotype of human tumor cells through different and not completely understood mechanisms. Specifically, *HGMA2* was regarded as a possible determinant of resistance to doxorubicin in liver cancer cells [32], to 5-fluorouracil in colorectal cancer cells [33], to docetaxel in gastric cancer cells [34] as well as to cisplatin in ovarian cancer cells [35]. In this context, we recently reported that the induction of *HMGA2* expression sustained the activation of a cytoprotective autophagic response in PDX and cell line models of epithelial sarcoma after treatment with the EZH2 inhibitor EPZ-011989 [25]. However, in the present study we only observed a modest increase in the expression of autophagy-related markers LC3B-II in one PDX models after treatment with selinexor or doxorubicin.

Consistent with previous evidence, it can be hypothesized that HMGA2 counteracts the effects of selinexor and doxorubicin in our DDLPS models by up-regulating AKT [36] and WNT [37] pro-survival pathways. It is also plausible that HMGA2 protects DDLP PDXs against the antitumor activity of anthracycline by promoting DNA double-strand break repair as a consequence of its documented ability to activate DNA damage response kinases ATM [38] and ATR [39].

XPO1 is the sole nuclear exporter for some of the major tumor suppressors (i.e., p53), cell cycle regulators (i.e., p21/CDKN1A) and growth promoting/anti-apoptotic proteins (i.e., survivin). The superior activity of selinexor compared to doxorubin observed in this study seems to be sustained by a greater ability to induce an apoptotic response in both PDXs and cell lines. Consistently with previous reports [22, 40], apoptosis was found to be dependent on nuclear accumulation of p53. In addition, selinexor caused marked down-regulation of survivin, with an almost complete abrogation of the protein cytoplasmic pool, which is known to be responsible for survivin anti-apoptotic function [19]. Survivin down-regulation was already reported by us and others in experimental models of different human tumor types exposed to selinexor [22, 41, 42]and also observed in a DDLPS cell line [11]. Survivin plays a vital role in oncogenesis being involved in both cell cycle control and resistance to apoptosis [43]. As regards liposarcomas, it has been shown that survivin is over-expressed in pleomorphic liposarcoma clinical specimens [44] and that the protein is essential for the growth of myxoid liposarcoma cell lines [45]. Although no data are currently available on DDLPS, results from our study suggest an important role for the protein also in this liposarcoma subtype.

Concerning the possible mechanisms responsible for survivin down-regulation after exposure to selinexor, it was previously shown that, in triple negative breast cancer cells, XPO1 inhibition represses *survivin/BIRC5* transcription by inhibiting STAT3 acetylation and blocking STAT3 binding to the gene promoter [20]. According to previous evidence that forced retention of survivin in the nucleus promotes its clearance by the ubiquitin-proteasome pathways [21], we reported that selinexor induced the ubiquitination of the protein nuclear fraction in diffuse malignant peritoneal mesothelioma cells [22]. Interestingly, in the present study we provide evidence that both mechanisms concur to the down-regulation of survivin following selinexor treatment in DDLPS cells.

Although superior to doxorubicin, the antitumor activity of selinexor appears moderate in our PDXs. A limitation of our models is related to their histological characteristics that cover only the myogenic and rhabdomyoblastic heterologous differentiation of DDLPS, which can present also other dedifferentiation subtypes, such as pleomorphic and epithelioid differentiation. Additional models of DDLPS expressing other differentiation lineages are currently under development in our lab to investigate if the activity of several anti-cancer drugs, including doxorubicin and selinexor, can differ across heterologous dedifferentiation subtype.

On the other side, the good tolerability of selinexor supports a try with combination regimens aimed at increasing its therapeutic effect. On this basis we started to explore the antitumor effect of selinexor combined with doxorubicin, a regimen that is currently under investigation in a prospective phase 1b clinical study of advanced soft-tissue sarcomas (ClinicalTrials.gov Identifier: NCT03042819). Among the 24 evaluable patients with different sarcoma types (only 2 LPS were included) who entered the trial, 5 (21 %) had a partial response by RECIST as best response [46]. In a preliminary experiment on the LS-GD-1 PDX model, we found that concomitant exposure with doxorubicin did not increase the antitumor activity of the XPO1 inhibitor (Max TVI: 82 % vs. 80 %). Of course, we cannot exclude that different treatment schedules could improve the result. It is also conceivable that combination regimens including drugs able to target specific XPO1 cargo proteins could result in a better therapeutic performance.

## Conclusions

Overall, results from this study indicate that, although moderate, the antitumor activity of selinexor is consistently higher than doxorubicin, irrespective of the extent of MDM2 amplification and the heterologous differentiation of the PDX models. Mechanistically, the nuclear accumulation of p53 and the depletion of survivin mainly contribute to the induction of apoptosis through which selinexor exerts its antitumor activity. Moreover, the good tolerability of selinexor supports a try with combination regimens aimed at increasing its therapeutic effect.

## Data Availability

The dataset and materials using during the current study are available from the corresponding author on reasonable request.

## Abbreviations

DD: Dedifferentiated
DDLPS: Dedifferentiated liposarcoma
ddPCR: Droplet digital PCR
FFPE: formalin-fixed paraffin-embedded
FNCLCC: Fédération Nationale des Centres de Lutte Contre Le Cancer
FISH: Fluorescence in situ hybridization
LPS: Liposarcoma
PDX: Patient- erived xenograft
TV: Tumor volume
TVI: Tumor volume inhibition
WD: Well differentiated
WDLPS: Well differentiated liposarcoma
